# 用于富集低分子量糖蛋白的多功能磁性纳米材料的制备

**DOI:** 10.3724/SP.J.1123.2021.07019

**Published:** 2021-10-08

**Authors:** Peng DOU, Yumiao XIANG, Liang LIANG, Zhen LIU

**Affiliations:** 南京大学化学化工学院, 生命分析化学国家重点实验室, 江苏 南京 210023; State Key Laboratory of Analytical Chemistry for Life Science, School of Chemistry and Chemical Engineering, Nanjing University, Nanjing 210023, China; 南京大学化学化工学院, 生命分析化学国家重点实验室, 江苏 南京 210023; State Key Laboratory of Analytical Chemistry for Life Science, School of Chemistry and Chemical Engineering, Nanjing University, Nanjing 210023, China; 南京大学化学化工学院, 生命分析化学国家重点实验室, 江苏 南京 210023; State Key Laboratory of Analytical Chemistry for Life Science, School of Chemistry and Chemical Engineering, Nanjing University, Nanjing 210023, China; 南京大学化学化工学院, 生命分析化学国家重点实验室, 江苏 南京 210023; State Key Laboratory of Analytical Chemistry for Life Science, School of Chemistry and Chemical Engineering, Nanjing University, Nanjing 210023, China

**Keywords:** 硼亲和, 糖蛋白, 分子识别, 纳米材料, 尺寸排阻, boronate affinity, glycoproteins, molecular recognition, nanoparticles, size exclusion

## Abstract

低分子量糖蛋白被认为是发现疾病生物标志物的宝库。特异性的萃取吸附剂对这一类化合物的萃取和富集是必不可少的。硼亲和材料在近年来取得了很大的发展,但专门用于选择性富集低分子量糖蛋白的硼亲和材料目前鲜有报道。该文提出了具有多种功能的磁性纳米颗粒(MNPs),用于低分子量糖蛋白的选择性捕获。该多功能磁性纳米颗粒是用硼酸功能化聚合物网络包裹的磁性纳米复合物。该多功能磁性纳米材料是利用磁性的纳米颗粒内核通过在其表面修饰苯硼酸功能团的聚丙烯酸高分子网络链制备得到。该材料不仅具有常规磁性材料在磁分离方面的基本优势,还能提供三重预先设计的先进功能:1)尺寸排阻效应,去除高分子量蛋白质的干扰;2)对低分子量糖蛋白的选择性萃取;3)保护捕获到的低分子量糖蛋白不被降解和污染。该材料的选择性萃取功能来自于硼酸配基与糖蛋白的顺式二醇部分的亲和性,而尺寸限制效应和保护功能则依赖于磁性纳米颗粒表面修饰的聚合物网络,允许低分子量化合物选择性通过。通过实验验证了这些预设的功能,且通过改变聚合物链长可以调节限径效应的阈值。这种多功能磁性纳米复合物可以进一步发展成有前景的纳米探针,不仅可以选择性捕获低分子量糖蛋白,还可以选择性捕获核苷和聚糖等其他具有重要生物学意义的顺式二醇分子。因此,该文报道的材料制备策略为从复杂样品中选择性萃取靶标化合物的多功能吸附剂的设计和合成提供了新思路。

## Abstract (en)

Low-molecular-weight glycoproteins (LMW-GPs) are considered promising candidates for disease biomarker discovery. Selective sorbents are essential for the extraction and enrichment of this class of compounds. Boronate affinity chromatography is a unique separation mode in liquid chromatography. It enables the selective separation and isolation of *cis*-diol-containing compounds such as glycoproteins and saccharides. Recent years have witnessed the rapid development of boronate affinity materials, particularly for use as selective sorbents in proteomics and metabolomics. However, studies are scarce on the specific design of such materials for the selective extraction of LMW-GPs. Herein, we present multifunctional magnetic nanoparticles (MNPs) for selectively harvesting LWM-GPs. The multifunctional MNPs were rationally designed and prepared by wrapping magnetic core nanoparticles with a phenylboronic acid-grafted poly(acrylic acid) (PAA) network. In addition to fulfilling the primary function of conventional MNPs in magnetic separation, multifunctional MNPs can offer three pre-determined advanced functions: 1) the size-restriction effect, which enables the elimination of the interference of high-molecular-weight proteins and other species; 2) the selective extraction of LMW-GPs; and 3) protection of the harvested LMW-GPs against degradation and contamination. The multifunctional MNPs enable selective extraction due to the affinity of the boronic acid ligand to the *cis*-diol moieties of the glycoproteins. The size-restriction effect and protection function depend on the polymer network on the surface of the MNPs, which allows the selective passage of low-molecular-weight molecules. Transmission electron microscopy (TEM) characterization showed that the MNPs were well-shaped nanoparticles, with a diameter of approximately 60 nm. The size-restriction effect was first predicted by a thermogravimetric analysis-based theoretical calculation, where for MNPs prepared using PAA with an average molecular weight of 240 kDa, the estimated pore size of the network was 0.9 nm. The boronate affinity and size-exclusion effect were verified by sodium dodecyl sulfate-polyacrylamide gel electrophoresis (SDS-PAGE) and capillary zone electrophoresis (CZE). To investigate the dependence of the selectivity of the MNPs to LMW-GPs in a complex environment and the size-restriction threshold for the PAA chain length, nano-liquid chromatography-tandem mass spectrometry (nano-LC-MS/MS) was performed to analyze the molecular mass of fragments harvested by the MNPs from the tryptic digest of horseradish peroxidase (HRP, a typical glycoprotein). The polymer chain length or the molecular weight of the PAA used played a critical role in determining the molecular weight thresholds of proteins above which the size exclusion effect will occur. The threshold values were found to be 5.0, 9.3, 4.1, 5.1, and 2.7 kDa for MNPs prepared using PAA with average molecular weights of 2, 5, 15, 100, and 240 kDa, respectively. This dependence enabled adjustment of the threshold value for inducing the size-exclusion effect of the multifunctional MNPs by changing the PAA chain length. The multifunctional MNPs can be further developed into promising nanoprobes for selectively harvesting not only LMW-GPs, but also other *cis*-diol-containing biomolecules of biological importance, such as nucleosides and glycans. Thus, the material preparation strategy reported herein offers new insights for the rational design and synthesis of multifunctional-affinity sorbents to selectively extract target compounds from a complex sample matrix.

蛋白糖基化是生物体中普遍发生且重要的生物学过程,其参与多种分子生物学的功能和途径^[1,2]^,是临床诊断重要的生物标志物。研究表明,体液中低分子量蛋白质(LMWP)和多肽含有丰富的生物学信息,能从中发现大量的生物标志物^[3]^。因此,低分子量糖蛋白质(LMW-GP)是生物标志物研究的重点。然而,低分子量糖蛋白在生物样品中丰度很低,糖肽/糖蛋白在质谱检测时容易被其他非糖蛋白抑制,因此需要发展高效的低糖蛋白分离富集方法。

对于富集低分子量蛋白质,目前已有的方法包括超滤^[4]^、尺寸排阻色谱^[5,6]^、尺寸限制性材料萃取^[7,8]^,基于纳米孔道的膜或颗粒的萃取也发展成一种新的替代方案^[9,10]^。近期,纳米材料在富集低丰度生物标志物方面具有巨大的潜力^[11]^。目前糖蛋白的分离富集方法主要有凝集素亲和色谱法^[12,13,14]^、固相肼化学富集方法^[15,16]^、磁性纳米材料硼亲和萃取^[17,18,19]^、硼亲和色谱^[20,21]^、亲水相互作用色谱法^[22,23]^等。然而,就作者所知,目前还没有专门用来富集低分子量糖蛋白的方法。

硼亲和材料在近年来取得了很大的发展^[19]^。特别是,将功能化磁性纳米材料(MNPs)作为微萃取探针用于选择性富集生物样品的研究越来越受到科研工作者的重视^[24,25,26,27,28]^。相比于常规方法,磁性纳米材料的优势是比表面积大,萃取容量高,可操控性强,生物兼容性好。同时,硼酸已经成为很有前途的特异性富集糖蛋白的亲和配体^[20,21]^。硼酸亲和法的原理为:硼酸分子与含顺式二羟基结构的化合物在碱性条件下形成五、六元环脂的复合物结构,而在酸性条件下,该复合物发生水解,从而释放出靶标分子。和凝集素亲和色谱相比,硼亲和色谱具有以下的优势:首先,硼亲和色谱对糖蛋白具有广泛的选择性,不仅可以富集*N*-糖蛋白,也可以富集*O*-糖蛋白;其次,硼亲和色谱可以通过简单地改变环境pH值来控制糖蛋白的捕获和释放,最后,硼亲和色谱可以通过挥发性酸性溶液洗脱被萃取的物质,因此是完全质谱兼容的,但专门用于选择性富集低分子量糖蛋白的硼亲和材料目前鲜有报道。

本文报道一种可以选择性富集低分子量糖蛋白的多功能磁性纳米材料。该多功能磁性纳米材料有特殊的外部结构,它的表面修饰了硼酸功能团高分子网络链,除了具有磁性纳米材料的普遍特性外,该纳米粒子具有如下3个特性:1)尺寸排阻性能,消除高分子量蛋白质和其他物种的干扰;2)特异性萃取低分子量糖蛋白,3)保护萃取到的低分子量的糖蛋白不被酶解和污染。多功能磁性纳米材料的结构和尺寸排阻原理如图1所示。磁性纳米材料的表面修饰了聚丙烯酸高分子网络,缠绕的线性聚合物链覆盖在磁性纳米材料的表面,形成了一个尺寸排阻的网络链,使得只有低分子量的分子才能通过。聚丙烯酸(PAA)链上的聚丙烯酸的部分羧基上键合了苯硼酸配基,因为羧基具有亲水性,而苯基硼酸具有一定的疏水性,在水溶液中,高分子链上的羧基处于高分子网络外侧保证表面很好的亲水性,而苯硼酸基团相对疏水,处于高分子网络内侧,因此只有通过高分子网络的低分子量分子中的糖蛋白才能被特异性萃取,另外,被捕获的低分子量的糖蛋白由于处于高分子网络内部,高分子网络的空间位阻使得低分子量糖蛋白周围没有适合酶相互作用的空间,因此起到了保护富集到的低分子量糖蛋白不受酶解的作用。本文对该材料的结构和性能进行了表征和验证,并考察了PAA链的分子量对材料的尺寸排阻效应的影响。

**图1:**
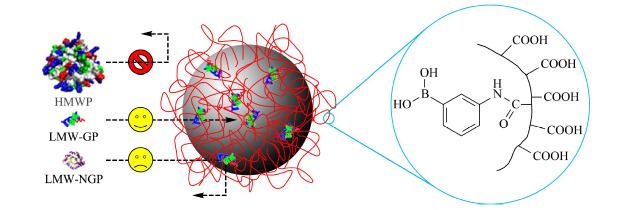
间氨基苯硼酸嫁接聚丙烯酸链修饰的磁性纳米颗粒(APBA-PAA-MNPs)的尺寸排阻效应和聚合物链的结构示意图

## 1 实验部分

### 1.1 仪器、试剂、材料和方法

*N*-羟基琥珀酰亚胺(NHS)购自阿拉丁试剂公司(上海)。牛血清白蛋白(BSA)、辣根过氧化物酶(HRP)和平均分子质量为100 kDa、15 kDa、5 kDa、2.1 kDa的PAA购自美国Sigma公司。丙烯酰胺/甲叉双丙烯酰胺预混液(*C*=2.6%)、四甲基乙二胺(TEMED)和溴酚蓝购自美国Bio-Rad公司。1-乙基-(3-二甲基氨基丙基)碳酰二亚胺(EDC)、间氨基苯硼酸(APBA)和平均分子质量240 kDa的PAA购自Alfa Aesar公司(天津)。十二烷基硫酸钠-聚丙烯酰胺凝胶电泳(SDS-PAGE)相对分子质量标准品购自Promega公司(上海),胰蛋白酶购自Promega (北京)。其他试剂购自南京化学试剂公司,均为分析纯。HPR酶解方法参照文献^[29]^,将50 μg HRP与2 μg胰蛋白酶混合溶于25 μL 50 mmol/L碳酸氢铵溶液中,在37 ℃下酶解过夜,产物存放于-20 ℃冰箱中。

材料的形貌表征采用日本JEOL公司的JEM-1010型透射电子显微镜,加速电压100 kV。制样是将纳米材料均匀分散在水中后滴在铜网上,自然风干备用。红外光谱为美国Thermo Fisher公司的Nicolet 6700型傅里叶变换红外光谱仪。Zeta电位测定采用英国Malvern公司的Nano Z zeta电位仪,测定时磁性纳米材料被分散在纯水中。SDS-PAGE采用美国Bio-Rad公司的Mini Protean 3电泳槽,电源型号为Powerpac HV。电泳结束后采用考马斯亮蓝R-250染料(购自美国Bio-Rad公司)进行染色,并用Gel Doc XR documentation凝胶成像系统进行记录。基质辅助激光诱导解离-飞行时间质谱(MALDI-TOF MS)在德国Brucker公司的Autoflex质谱仪上完成。纳流液相色谱-质谱联用(nano-LC-MS)实验在美国Thermo Fisher公司的纳流LC-LTQ-Orbitrap XL系统上完成。毛细管电泳实验在美国Beckman Coulter公司的P/ACE MDQ毛细管电泳仪上完成。

### 1.2 功能化磁性纳米材料合成

1.2.1 氨基功能化磁性纳米材料(AMNPs)^[30]^

将1.0 g六水合三氯化铁、6.5 g 1,6-己二胺和2.0 g无水碳酸钠溶于30 mL乙二醇中,装入带有四氟乙烯内衬的50 mL高压水热釜中在198 ℃下反应6 h。制得的氨基功能化磁性纳米材料用水和乙醇各清洗3次后在50 ℃下真空烘干备用。

1.2.2 多功能磁性纳米材料

将500 mg AMNPs分散于50 mL 30 g/L的PAA溶液中,并加入500 mg EDC和1 g NHS。超声混合均匀,机械搅拌2 h。制得的PAA活化磁性纳米材料(PAA-grafted AMNPs)用水和乙醇各清洗3次后在50 ℃下真空烘干备用。取200 mg PAA-grafted AMNPs分散在40 mL 5 g/L间氨基苯硼酸一水合物溶液中,并加入400 mg EDC和800 g NHS。超声混合均匀,机械搅拌2 h。制得的多功能磁性纳米材料(APBA-PAA-MNPs)用水和乙醇各清洗3次后在50 ℃下真空烘干备用。

1.2.3 间氨基硼酸功能化磁性纳米材料

将400 mg AMNPs分散于40 mL含5% (v/v)戊二醛的100 mmol/L磷酸钠缓冲溶液(pH 7.0)中机械搅拌2 h。得到的戊二醛活化的磁性纳米材料用100 mmol/L磷酸钠缓冲溶液清洗3次后分散于含40 mL 5 g/L间氨基苯硼酸一水合物和1%(质量分数)的氰基硼氢化钠的100 mmol/L磷酸钠缓冲溶液中。反应2 h后,收集制得的间氨基硼酸功能化磁性纳米材料(APBA-GA-MNPs)用水和乙醇各清洗3次后在50 ℃下真空烘干备用。

### 1.3 萃取和解吸

SDS-PAGE分析 将HRP和BSA溶解在含500 mmol/L NaCl的50 mmol/L NH_4_HCO_3_-NH_3_·H_2_O(pH 10)中,每种蛋白质各1 g/L。取2 mg MNPs和50 μL上述蛋白质溶液,在一个PCR管中混匀,振荡萃取1 h。随后将MNPs用外加磁场吸附于管壁,弃去上清液。MNPs用含500 mmol/L NaCl的50 mmol/L NH_4_HCO_3_-NH_3_·H_2_O(pH 10)和50 mmol/L NH_4_HCO_3_-NH_3_·H_2_O(pH 10)各清洗3次后分散于25 μL 50 mmol/L醋酸中振荡解吸1 h。最后将MNPs用外加磁场吸附于管壁,收集解吸后的醋酸溶液准备进一步分析。

毛细管电泳分析 萃取解吸步骤相同,唯一不同的是在萃取用的HRP、BSA混合溶液中加入了1 g/L腺苷。

nano-LC-MS分析 将HRP酶解产物与含1 mol/L NaCl的100 mmol/L NH_4_HCO_3_-NH_3_·H_2_O(pH 10)等体积混合后取50 μL用于萃取,其他步骤同上。

### 1.4 SDS-PAGE分析

SDS-PAGE分析采用1 mm厚度的凝胶。用于浓缩胶灌制的各种溶液的配比如下:15 mL 30%的丙烯酰胺/甲叉双丙烯酰胺预混液(*C*=2.6%), 6.3 mL 0.5 mol/L Tris-甘氨酸缓冲液(pH 6.8), 0.25 mL 10% SDS溶液,15 mL超纯水,0.125 mL 10%过硫酸铵和0.025 mL四甲基乙二胺(TEMED)。用于分离胶灌制的各种溶液的配比如下:4.0 mL 30%的丙烯酰胺/甲叉双丙烯酰胺预混液(*C*=2.6%), 2.5 mL 1.5 mol/L Tris-甘氨酸缓冲液(pH 8.8), 0.10 mL 10% SDS溶液,3.35 mL超纯水,0.050 mL 10%过硫酸铵和0.005 mL TEMED。制胶时采用10孔的制孔梳。样品与5×的上样缓冲液(100 g/L SDS、10 mmol/L二硫苏糖醇、20%(v/v)甘油、0.2 mol/L Tris-HCl (pH=6.8), 0.5 g/L溴酚蓝)按4;1(v/v)的比例混合,在100 ℃下加热3 min。等样品冷却到室温后取20 μL加入上样孔。在电泳过程中,前25 min电压为100 V,而后将电压升高到200 V直至溴酚蓝条带运行到凝胶底部。

### 1.5 毛细管电泳分析

实验中使用的毛细管为60 cm(有效长度50 cm)×75 μm I.D.×375 μm O.D.,购自河北永年光纤厂。每天实验开始前,均使用如下条件平衡毛细管:1 mol/L NaOH冲洗20 min,纯水冲洗20 min,背景缓冲溶液(BGE)冲洗20 min。每两次实验之间,用以下条件平衡毛细管:0.1 mol/L NaOH冲洗2 min,纯水冲洗2 min, BGE冲洗2 min。BGE组成为35 mmol/L硼砂缓冲液(pH 10.0)。毛细管温度控制在25 ℃。进样方式采用压力进样,进样压力3.45 kPa(0.5 psi),进样时间3 s。分离电压设定在20 kV。检测器的检测波长设定为214 nm。

### 1.6 Nano-LC-MS分析

LC-LTQ-Orbitrap XL液相色谱-质谱联用系统配有两台四元梯度泵,一个微量自动进样器和配有纳喷源的LTQ-Orbitrap XL高分辨质谱。实验中我们选用Agilent公司的富集柱(5 mm×0.3 mm,填料为Zorbax 300SB-C18 5 μm)。分析柱为自制的填装C18毛细管色谱柱,内径75 μm,长度12 cm。制作方法简述如下:首先在一段石英毛细管(75 μm I.D.)的一端用丁烷火焰烧制拉出一个内径5~10 μm的喷针;随后采用自制的压力容器将C18 AQ反向填料匀浆(粒径5 μm,孔径12 nm,购自德国Sunchrom公司)装填到拉制好的毛细管中,装填压力约4 MPa,在填料长度达到12 cm时停止装填。

流动相组成为(A)0.1%(v/v)甲酸水溶液,(B)0.1%(v/v)甲酸的乙腈溶液。实验中上样泵的流动相恒定为98%A,流速60 μL/min。连接质谱仪的色谱梯度如下:0~5 min, 2%B; 5~40 min, 2%B~60%B; 40~45 min, 60%B~90%B; 45~55 min, 90%B保持10 min; 55~75 min, 2%B。流动相在进入分析柱之前经过三通分流后的流速约为200 nL/min。质谱检测方法设定如下:喷雾电压设定为1.8 kV,每次扫描时,在Orbitrap中进行一个10万分辨率的全扫描,同时在LTQ中进行6个数据依赖性二级质谱扫描。每个全扫描和二级质谱扫描均含有一个micro scan。全扫描的质荷比(*m/z*)范围是400到2000,二级质谱扫描时仪器自动选择全扫描中丰度最高的6个离子进行碰撞诱导解离碎裂,归一化碰撞能量设定为35%。Orbitrap的目标离子强度设为2×10^5^,最大采集时间设为500 ms, LTQ的目标离子强度设为3×10^4^,最大采集时间设为100 ms。动态排除的设定方式如下:重复2次,重复时长30 s,排除时长60 s。

### 1.7 MALDI-TOF MS分析

MALDI-TOF MS分析在Autoflex MALDI-TOF/TOF质谱仪上完成。实验采用正离子模式,激光波长为337 nm,脉冲时间250 ns,加速电压20 kV。实验中使用不锈钢靶,基质为*α*-氰-4-羟基肉桂酸(CHCA)的饱和溶液,溶剂为0.1%(v/v)三氟乙酸-乙腈(70;30, v/v)。每张谱图由100次激光发射产生的数据叠加而得。

## 2 结果与讨论

### 2.1 纳米材料表征

我们采用透射电子显微镜对合成的APBA-PAA-MNPs纳米材料的形貌进行了表征。如图2a所示,该材料的形貌完好,粒径均匀,平均直径为60±15 nm。随后,我们通过红外光谱表征了表面的官能团(见图2b),在3种纳米材料的红外光谱中,在576 cm^-1^处均有一个很强的吸收峰,对应的是Fe-O键的振动峰。同时在2855 cm^-1^和2927 cm^-1^处的双峰则对应着-CH_2_-官能团。对AMNPs,1623 cm^-1^和1048 cm^-1^处的双峰则对应了C-NH_2_的振动峰,说明磁核纳米材料确实被-NH_2_ 功能化了。对PAA-grafted AMNPs,1407 cm^-1^处的峰证实了羧基的存在,1456 cm^-1^和1715 cm^-1^处的峰则对应着酰胺基团,说明PAA确实是共价地连接到了纳米材料表面。对APBA-PAA-MNPs,1811、1783和1590 cm^-1^处的峰说明材料上有苯环存在,而1208 cm^-1^处的峰则对应-C-N-苯基团,这些都说明材料上已经连接了间氨基苯硼酸。

**图2:**
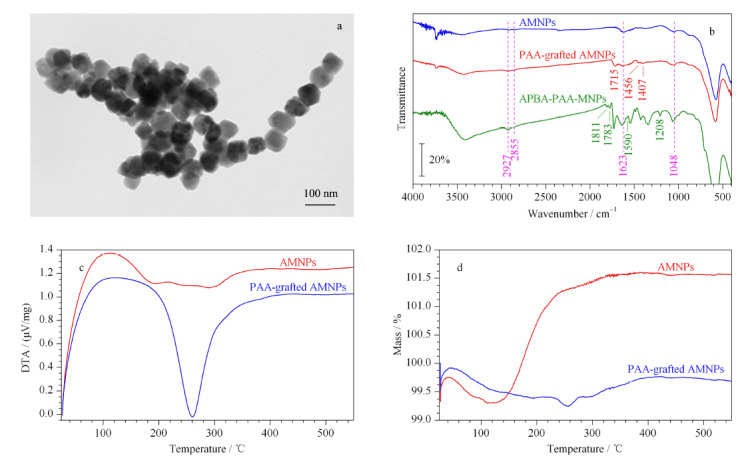
纳米材料的表征

我们测量了Zeta电位的变化。AMNPs的Zeta电位为+2 mV,修饰了240 kDa PAA的MNPs的zeta电位为-25 mV,而修饰了间氨基苯硼酸的MNPs的Zeta电位为-20 mV。Zeta电位由+2 mV变为-25 mV,说明材料表面被大量PAA覆盖,而Zeta电位由-25 mV变为-20 mV,说明只有部分的羧基连接了间氨基苯硼酸。

我们估算了PAA网络的孔径和最大萃取容量。MNPs的密度约为5 g/cm^3[31]^,由于前面的电镜表征显示其直径约为60 nm,可以计算出每个纳米颗粒的平均质量约为4.5×10^-15^ g。差热分析结果显示,PAA链在260 ℃时燃烧(见图2c)。进一步的热重分析(见图2d)显示,PAA链燃烧后导致的质量损失占总质量的2%。这表明,在该PAA链修饰的MNPs上的高分子链的质量约占总质量的2%,因此可以计算得到,每个纳米颗粒上的高分子链总质量平均约为1.1×10^-17^ g。此处PAA链的平均分子质量为240 kDa,则每条PAA链的质量约为4.0×10^-19^ g,因此每个磁性纳米颗粒上大约接有25条PAA(240 kDa)链。另外,根据分子量可以计算出PAA链的聚合度约为3300,而C-C键键长约为0.15 nm,因此可知每条PAA链的平均长度约为990 nm。我们假定PAA链可以均匀包裹在磁性纳米颗粒上,则由高分子构建出来的网络的孔径可以估算得到大约为0.9 nm。由此,我们可以预期由PAA网络覆盖的MNPs具有尺寸排阻效应。由于在实际情况下高分子链是在水溶液环境中使用的,而且其空间排布上是相对松散的,因此其孔径可能更大一些。因此,实际的尺寸排阻效应的分子量阀值应以实验结果为准。

### 2.2 多功能磁性纳米材料的选择性和尺寸排阻性能表征

以APBA-GA-MNPs做对照,我们利用SDS-PAGE表征了APBA-PAA-MNPs的尺寸排阻性能。HRP和BSA分别作为糖蛋白和非糖蛋白的代表用于表征。如图3所示,APBA-GA-MNPs可以选择性地从HRP和BSA的混合物中萃取HRP,而APBA-PAA-MNPs则对两种蛋白质均不结合。该结果表明,由于HRP(分子质量44 kDa)的尺寸大于APBA-PAA-MNPs材料表面高分子网络形成的孔的直径,因此无法被APBA-PAA-MNPs富集。

**图3:**
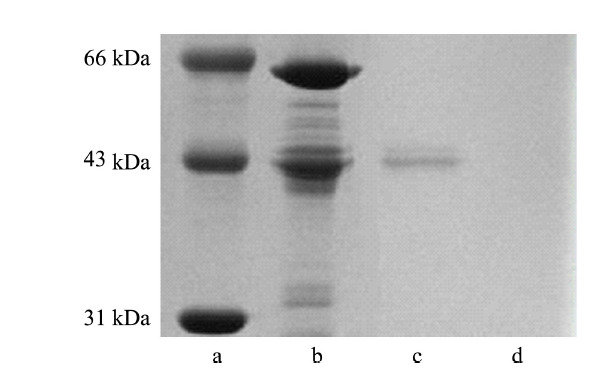
选择性和尺寸排阻效应的SDS-PAGE表征

为了进一步确证以上结论,将小分子顺式二醇化合物腺苷(分子质量267 Da)与HRP和BSA混合后得到的溶液作为样品,经不同的材料萃取后得到的化合物用毛细管电泳进行分析。如图4所示,APBA-PAA-MNPs材料只能捕获腺苷,而APBA-GA-MNPs材料则可以同时捕获腺苷和HRP。由此就可以得出结论,APBA-PAA-MNPs材料具有硼亲和尺寸排阻双重选择性。

**图4:**
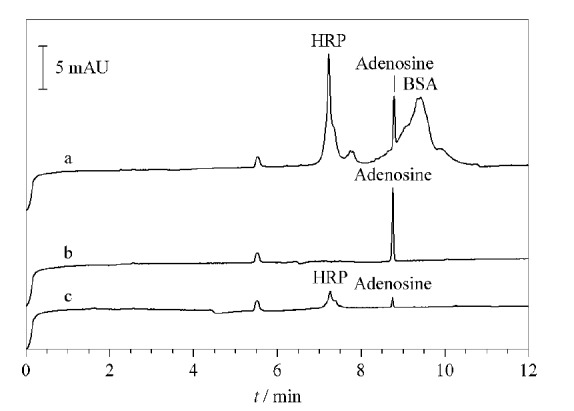
选择性和尺寸排阻效应的毛细管电泳表征

### 2.3 多功能磁性纳米材料的特异性和尺寸排阻性能与PPA链长的关系

为了考察APBA-PAA-MNPs在复杂环境中对低分子量糖蛋白的富集能力以及尺寸排阻阈值和PAA链长的关系,我们用nano-LC-MS/MS分析了不同链长PAA修饰的APBA-PAA-MNPs从HRP酶解产物中富集到的肽段。实验中通过二级质谱中的特征离子和糖基的中性丢失来确定富集到的肽段是否为糖肽^[32]^,通过如下步骤对得到的二级质谱图进行分析:首先确定哪个时间段得到的谱图中含有*m/z*为366 ([Hex_1_HexNAc_1_+H]^+^)、528 ([Hex_2_HexNAc_1_+H]^+^)、690 ([Hex_3_HexNAc_1_+H]^+^)和822 ([Hex_2_Pent_1_HexNAc_1_+H]^+^)的特征离子,如果谱图中存在这些离子则认为是糖肽的二级质谱;当确定了糖肽的出峰时间窗口之后,将整个时间段内的所有全扫描质谱图用Qual Browser功能进行平均,然后用Xtract功能做去卷积,将所有多电荷离子转化为MH^+^形式,Xtract中*S/N*设为10。单糖残基的中性丢失见表1,如果某个肽段的二级质谱图中含有至少一个上述的特征离子,并有一系列规律的中性丢失则可认为其为糖肽,图5中以MH^+^=2542.15 Da的糖肽为例标明了各个特征离子和中性丢失。

**表1:** 寡糖中性丢失造成的质量减少

Sugar	Neutral loss under different charge states		
	+1	+2	+3
Hexose	162.1	81.0	54.0
Deoxyhexose	146.1	73.0	48.7
Pentose	132.0	66.0	44.0
N-Acetylhexosamine	203.1	101.6	67.7

**图5:**
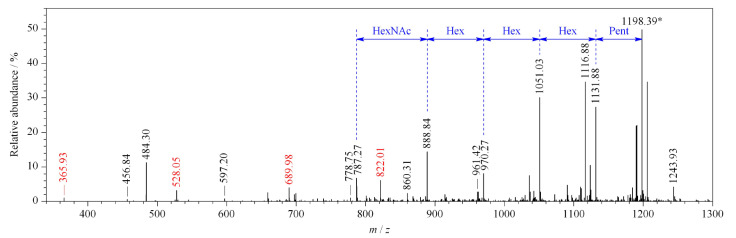
用于定性糖肽的特征离子的二级质谱图

由于绝大多数文献中报道的HRP酶解产物中糖肽的分子质量在1800到5000 Da之间^[22,23]^,因此我们首先考察了1800到5200 Da这个质量范围。图6中是不同PAA链长修饰的磁性纳米材料富集到的所有糖肽的去卷积质谱图。从HRP酶解糖肽中鉴定到了14个非糖肽段和35个糖肽段(见表2),而被APBA-PAA-MNPs富集到的肽段的数目要明显小于HRP酶解产物中的肽段数目。所有被APBA-PAA-MNPs富集到的肽段均为糖肽,非糖肽被全部排除在外,这说明APBA-PAA-MNPs在复杂体系中依然具有很高的选择性。用平均分子质量为5 kDa的PAA修饰的MNPs富集到的糖肽是所有材料中最多的,HRP酶解产物中76%的肽段被该材料富集。而其他4种分子质量的PAA修饰的MNPs富集到的肽段数目均不足HRP酶解产物中糖肽数目的50%(详见附表:http://www.chrom-China.com)。由图6可知,2、5、15、100和240 kDa PAA修饰的MNPs能结合到的最大糖肽的分子质量分别为4985.19、5067.08、4114.70、5067.08和2624.16 Da。因此,这些PAA修饰的MNPs的尺寸排阻阈值应比这些分子质量略高。

**图6:**
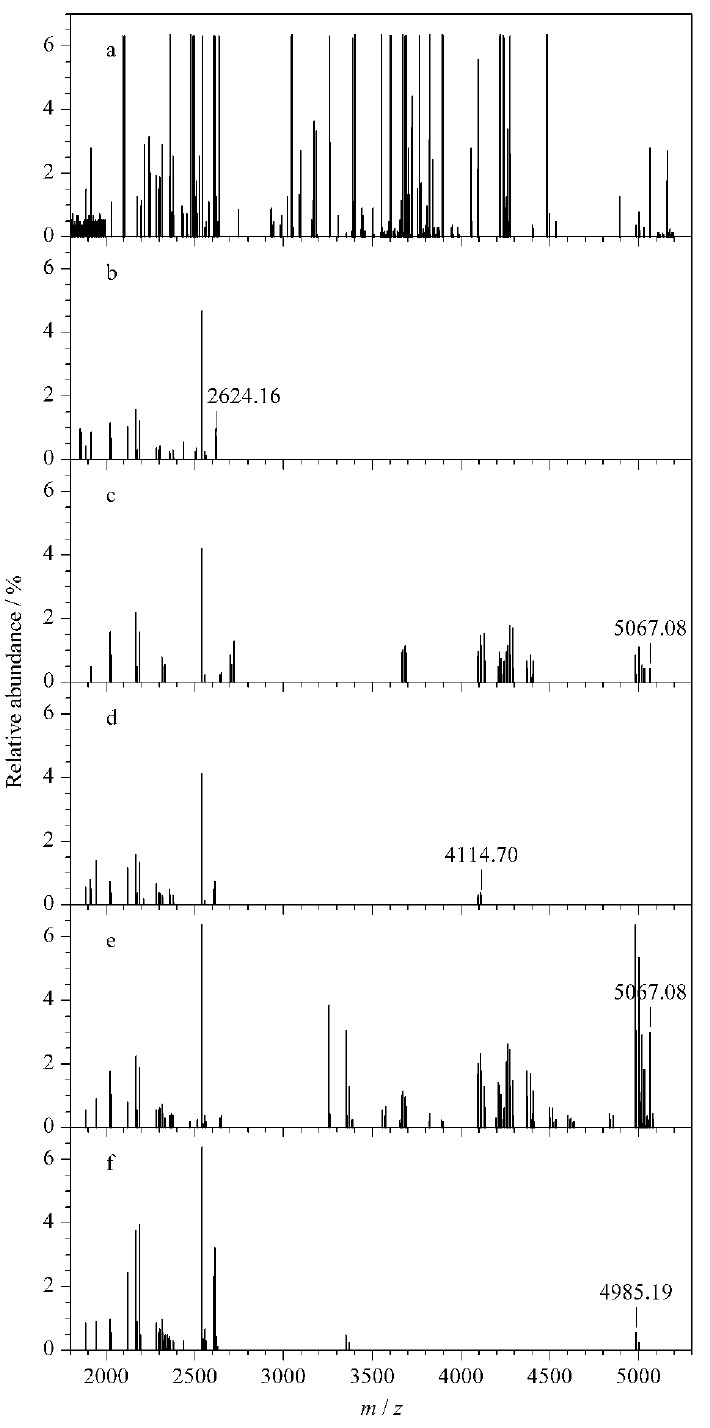
1800~5200 Da糖肽的去卷积质谱图

**表2:** HRP酶解糖肽中分子量在1800到5200 Da范围内鉴定到的非糖肽和糖肽

Type	Molecular mass/Da
Non-glycopeptides	2103.05, 2200.95, 2216.98, 2248.16, 2316.18, 2475.28, 2580.04, 2638.13, 2944.36, 2988.49, 3048.54, 3173.66, 3402.56, 3447.73
Glycopeptides	1887.84, 1916.74, 2029.82, 2173.88, 2286.98, 2363.01, 2436.23, 2542.615, 2612.20, 2932.24, 3021.20, 3095.30, 3184.27, 3257.52, 3354.41, 3390.46, 3553.52, 3591.65, 3606.62, 3673.71, 3726.63, 3753.65, 3766.64, 3776.73, 3825.67, 3840.65, 3895.66, 4057.71, 4114.70, 4223.85, 4276.77, 4482.99, 4895.19, 5067.08, 5165.36

另一方面,不同分子质量的PAA修饰的MNPs能够富集的最大的糖肽片段并不相同。由于有些糖肽段太过接近5200 Da的上限,我们进一步研究了分子质量高于5200 Da的肽段。通过MALDI-TOF MS实验发现,所有的APBA-PAA-MNPs均不能富集RNase B(糖蛋白,分子质量约为15 kDa)。因此我们进一步研究了nano-LC-MS/MS谱图中分子质量从5200到15000 Da的部分。由2、15、100、240 kDa PAA修饰的MNPs均无法富集任何肽段,仅有5 kDa PAA修饰的MNPs富集到了少量肽段,其中分子质量最大为9218.09 Da(见图7)。经过二级质谱鉴定,该肽段为糖肽。由于HRP酶解产物中有大于10 kDa的糖肽段,我们认为,2、5、15、100和240 kDa PAA修饰的MNPs的尺寸排阻阈值约为5.0、9.3、4.1、5.1和2.7 kDa。

**图7:**
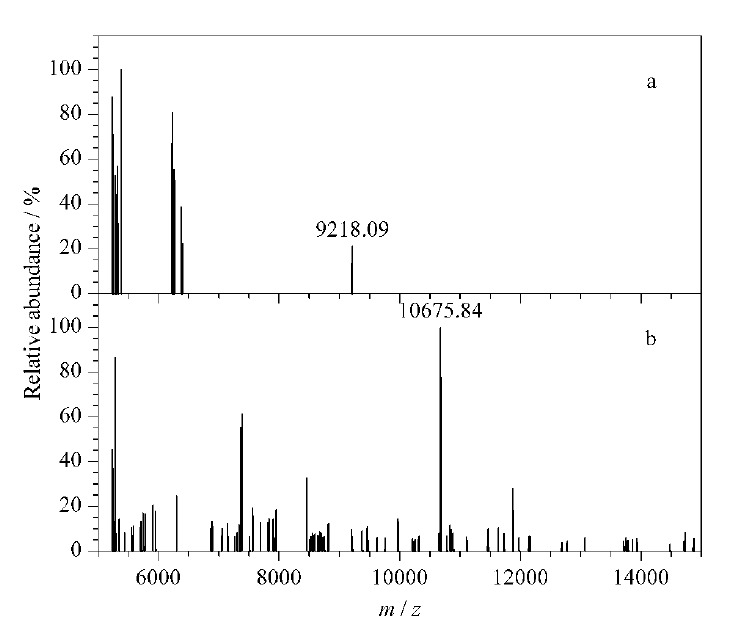
5200~15000 Da糖肽的去卷积质谱图

因此,可以得出结论:可以通过调节PAA链长来调节多功能MNPs的尺寸排阻阈值。

## 3 结论

本文提出了多功能磁性纳米材料的概念并证明了其原理,由于其特殊设计的结构,这种磁性纳米材料可以成为高选择性萃取低分子量的糖蛋白的多功能纳米探针,尽管研究中主要测试的分子是低分子糖蛋白,但是这种多功能磁性纳米材料同样可以选择性萃取其他低分子量顺式二羟基生物分子,如修饰核苷、多糖等,由于糖蛋白、核苷和聚糖是蛋白质组学、代谢组学和糖组学领域关注的研究焦点。若能进一步采用高亲和力配基,这种多功能磁性纳米材料可以发展成为一种很有前途的组学研究样品处理材料。
